# Selective embedment of silver nanocrystals into spatially segregated domains in thin polymer films for controlled fabrication of functional nanocomposites

**DOI:** 10.1039/c9ra02490e

**Published:** 2019-06-17

**Authors:** Michael Bushell, Anatoli Ianoul

**Affiliations:** Department of Chemistry, Carleton University 1125 Colonel By Dr Ottawa ON Canada anatoli.ianoul@carleton.ca +1-613-520-3749 +1-613-520-2600 ext. 6043

## Abstract

Fabrication of polymer-nanoparticle nanocomposites typically relies on mixing nanoparticle and polymer solutions, which renders little control over nanoparticle incorporation, and homogeneity of the resulting composite material. This work focuses on the thermally induced embedment of monocrystalline silver nanocubes (AgNCs) into polymer surfaces. The AgNCs are initially deposited through a Langmuir approach onto films of immiscible blended polymer films, which allows fine control over nanoparticle density and aggregation state. This nanoparticle/polymer composite is then heated above the glass transition temperature (*T*_g_) of a polymer, which initiates the irreversible embedding of the AgNCs. The immiscible ternary polymer films featured discrete domains (with different *T*_g_s), which were altered by changing the amount of polystyrene, poly(2-vinylpyridine) and poly(methyl methacrylate) within the polymer solution. The *T*_g_ dependence of the embedding process allowed the selective embedment of AgNCs into discrete polymer domains. The process was monitored in real time by using spatially separated hybrid plasmon modes, through peak shifts observed in a UV-vis spectrum. Enhanced surface confinement was observed for certain tripolymer films when compared to polystyrene–AgNC nanocomposites, due to changes in the surface energy within the blend. This work brings interesting insight on nanoparticle-blended polymer interactions and provides a fairly universal approach for the fabrication of these polymer–metal nanoparticle nanocomposites, which is of particular interest in fields that require fine control over nanoparticle incorporation within segregated polymer domains.

## Abstract 2

Thermally induced incorporation of plasmonic nanoparticles into specific polymer domains in a thin film is achieved and monitored in real time.

## Graphical abstract

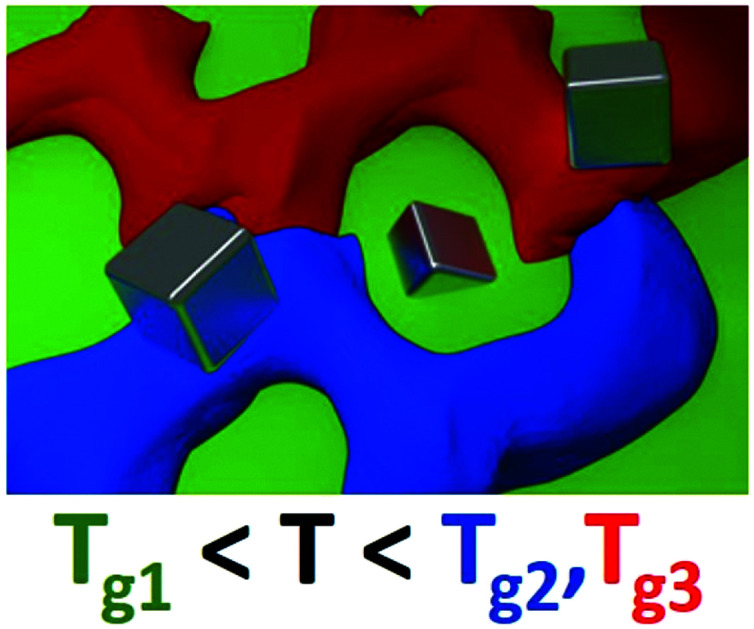

## Introduction

Polymer blends are of particular interest as they are a cost-effective means to optimize polymer material properties, such as: conductivity,^1^ bio-compatibility,^2^ rubberiness,^3^ and physical/chemical robustness.^4,5^ Incorporation of nanoparticles into polymer blends can even further enhance useful properties of these polymer blends, such as domain solubility and stability,^6^ and can lead to nanoparticle induced morphological changes.^7^ The optical properties of the nanoparticles can be tuned by changing the relative composition of the polymers within blend.^8^ Generally, metal or metal oxide nanoparticles are used, due to their advantageous electrical and optical properties. Ability to fabricate nanocomposite materials consisting of complex polymer mixtures and nanoparticles in a controlled and reliable way is of great importance.

Metal nanoparticles of gold and silver, which are of often used in nanocomposites due to their unique physico-chemical properties, can be incorporated into polymer films, through a thermally induced diffusion process.^9–13^ Two major constraints must be satisfied for the embedding to occur. The first is a kinetic requirement, which states that the polymer must be above its glass transition temperature (*T*_g_). This permits the necessary polymer re-arrangements enabling nanoparticle embedment, with higher temperatures providing greater mobility of the polymer; thus, faster embedding of the particle.^9,11^ The second requirement is a surface energy argument, which is always satisfied for bare metal nanoparticles^10^ and polyvinylpyrrolidone (PVP) capped silver nanocubes (as used in this work) embedding into polystyrene (PS), polyvinyl chloride and poly(methyl methacrylate) (PMMA) homopolymer films.^14^

Silver nanocubes (AgNCs) when supported on a dielectric substrate, such as a polymer film exhibit strong hybrid plasmon resonances that are spatially separated; thus, sensitive to different environments simultaneously.^15,16^ The hybrid dipolar plasmon (D) faces towards the area of highest refractive index (the polymer film) and the hybrid quadrupolar plasmon (Q) is situated near the area of lower refractive index (the surroundings).^17^ This anisotropy allows real-time sensing of the polymer–nanocube interface as the AgNCs embed into the polymer. The high refractive index sensitivity of the AgNCs enables accurate monitoring of nanocube embedding into the polymer surface. In our previous work we found that this high spatial sensitivity enables probing of a thin-surface layer above the bulk polymer, allowing the AgNCs to embed at temperatures below the bulk *T*_g_ of the polymer.^9^ Existence of this surface layer is in accordance with several other studies for ultra-thin polymer films.^18,19^ Knowledge of the temperature dependence of AgNCs embedding into homopolymers, allows the thermally activated selective embedment into discrete polymer domains to be initiated and controlled.

Immiscible ternary polymer blends are of interest as they possess segregated polymer domains with different polymer dynamics.^20,21^ In this work, a blend of polystyrene (PS), poly(2-vinylpyridine) (P2VP) and poly(methyl methacrylate) (PMMA) was used due to its ability to form tuneable well defined discrete domains, with different glass transition temperatures: 100 °C, 105 °C, and 118 °C for PS,^22^ P2VP^20^ and PMMA,^23^ respectively. AgNCs were deposited onto blended polymer thin films using a Langmuir technique for fine control over the density of the resulting nanocube monolayer. By heating the sample above the glass transition temperature, the AgNCs were irreversibly incorporated into the polymer. The process was monitored in real time allowing the fine control over AgNC embedment depth. The temperature specificity of the embedding process allows the selective embedment of AgNCs into individual polymer domains within the blended polymer film. Specifically, by simply heating the sample above the *T*_g_ of one polymer, only AgNCs on that polymer should embed.

The use of immiscible polymer blends enables great control over the morphology of the resulting film.^24–26^ Although polymer blends were used, this general concept can be further applied to any polymer with segregated domains, such as block copolymers, and various types of metal nanoparticles used instead of AgNCs. This work is significant for the production and characterization of useful hybrid nanoparticle-blended polymer materials.

## Experimental section

### Materials

Silver nitrate (99%+), polyvinylpyrrolidone (PVP, *M*_w_ ∼ 55 000), polystyrene (PS, *M*_w_ ∼ 192 000) polymethylmethacrylate (PMMA, *M*_w_ ∼ 120 000), anhydrous ethylene glycol (EG, 99.8%), sodium chloride (99%+), sodium sulfide (98%+), and mineral oil (heavy) were purchased from Sigma-Aldrich and used as obtained. Ethanol (95%) was obtained from Commercial Alcohols and used without further purification. Tetrahydrofuran (THF, 99.0%) was purchased through Caledon. Chloroform (99.5%+) was purchased through Alfa Aesar. Acetone (99.5%+) and methanol (99.8%+) were purchased from VWR International. Poly(2-vinylpyridine) (P2VP, *M*_w_ 200 000–400 000) was purchased from Polysciences Inc. 1,2-Dioleoyl-*sn-glycero*-3-phosphocholine (DOPC, powder) was purchased from Avanti Polar Lipids and used as obtained.

### Synthesis of silver nanocubes

All glassware was soaked in a solution of aqua regia (3 parts hydrochloric acid and 1 part nitric acid) for 30 minutes. Glassware was then rinsed; 52 times with tap water, 32 times with distilled (reverse osmosis) water, and 16 times with de-ionized water (18.2 MΩ cm) and dried in an oven. Silver nanocubes were synthesized according to a procedure described in literature.^27^ The reaction was quenched *via* an ice bath once the desired spectrum was observed with a dipolar resonance at ≈480 nm. Once cooled the solution was purified by centrifugation and stored in a refrigerator at 4 °C until use.

### Preparation of immiscible polymer blended thin films

Thin films were produced by spin coating blended polymer solutions on glass slides. Glass slides were cleaned by sonication in methanol, rinsing with acetone and with de-ionized water (18.2 MΩ cm), the slides were dried under rough vacuum at 110 °C. All blended polymer stock solutions were 3% w/w (total mass of polymer/mass of THF solvent). The polymer solutions were spun at ∼4000 RPM for 45 seconds using a homemade spin coater. The resulting films were allowed to rest at room temperature for 2 hours, after which they were annealed overnight at 140 °C. All cast polymer films were stored at room temperature and pressurized under nitrogen until use.

### Preparation of AgNC monolayers

Silver nanocrystals were deposited onto the polymer films by Langmuir–Schaefer deposition, as previously described.^16^ Specifically, a UV-vis spectrum of the AgNCs suspended in ethanol was obtained and used to determine the optimal aliquot of the AgNC solution to disperse in 250 μL of chloroform and 10 μL of a 1,2-dioleoyl-*sn-glycero*-3-phosphocholine solution (1 mg mL^−1^ in ethanol). The resulting solution was deposited onto the NIMA 311D trough filled with de-ionized water (18.2 MΩ cm). The monolayer was allowed to rest for 30 minutes for full chloroform evaporation. An isotherm was measured to obtain the desired uniform layer of silver nanocubes, which appear as a yellow film, and to determine the optimal pressure for deposition, typically, at the liquid–gas phase at about 0.3–0.7 mN per metre. AgNC monolayers were transferred by horizontally placing clean polymer substrates to the water surface. The resulting monolayer was dried using N_2_ gas. To determine quality of the monolayer, a UV-vis spectrum was obtained (Shimadzu, UV-2450).

### AgNC embedding experiments

Silver nanocube monolayers were heated using a homemade heating set up, designed to measure the temperature and UV-vis spectra automated, and in real-time. The setup was comprised of a heating pump (Thermo Fisher Scientific, RTE 7) pumping 95% EG into an aluminum heating block (Ocean Optics, CUV-ALL-UV cuvette holder). A xenon light source (Ocean Optics, PX-2) and detector (Ocean Optics, USB2000-UV-vis) were coupled to the heating block by use of optical fibers. A thermocouple (Vernier, TCA-BTA) was used to measure the temperature of the slide by direct attachment of the k-type thermocouple wire to the top of the sample. The time interval between spectral measurements was ∼ 3.4 seconds, 2500 accumulations were obtained for each heating experiment.

### Spectra processing

UV-vis spectra were processed and analyzed using OriginLab analysis software. Only the lower temperature heating experiments (attributed to the PS transition) were processed, to minimize peak de-convolution from the other polymers. Peak maxima were determined by batch peak processing using a first derivative fitting with a Savitzky–Golay (polynomial order 3, window size 25 points) smoothing to reduce baseline noise; this result was then compared to ∼500 randomly picked times to test validity.

### Topological measurements

Topography measurements were done using atomic force microscopy (AFM, Ntegra NTMDT, Russia, 100 × 100 μm scanner) in semi-contact (tapping) mode, 1024 × 1024 points per image, with silicon probe (135 μm long, 0.3–6 N m^−1^ spring constant (NSG03), resonance frequency 90 kHz, NT-MDT). AFM images were processed with Nova image processing software.

## Results and discussion

**Fig. 1:**
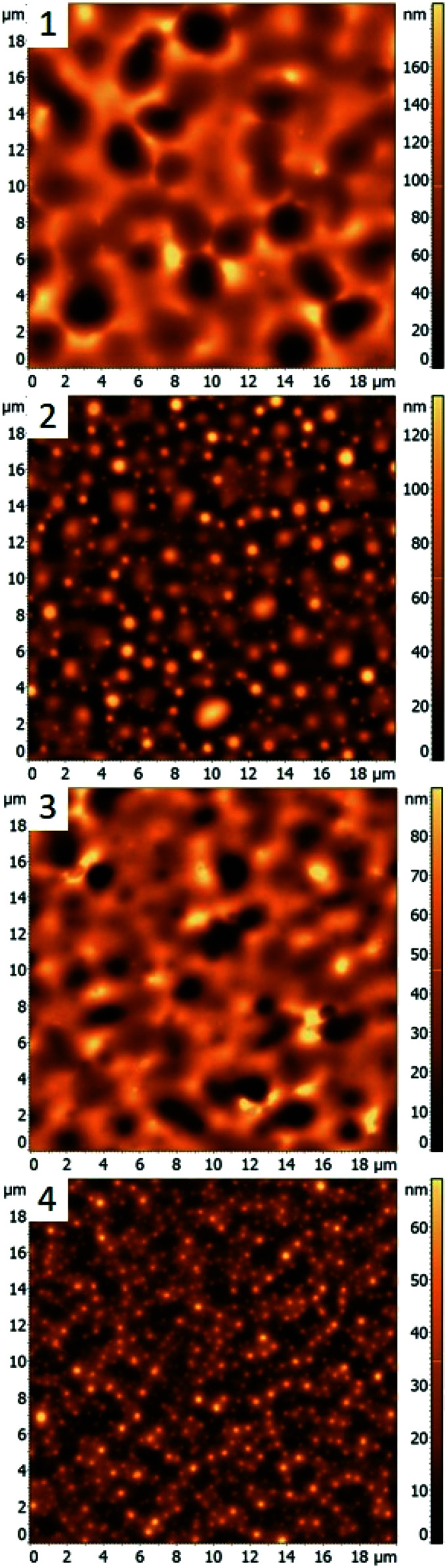
AFM images of blended thin films prepared using different PS/PMMA/P2VP compositions: (1) blend 1, with 34.5/31.0/34.5% composition; (2) blend 2, 48.7/23.1/28.2%; (3) blend 3 with 30.3/33.3/36.4%; (4) blend 4, with 60/20/20%.

In this work, different polymer blends were used to investigate a relationship between the nature of polymer films and properties of nanocomposite materials obtained by incorporating silver nanocrystals into such films. Blends of three immiscible polymers PS, PMMA and P2VP were used to prepare thin films with well-separated domains of the individual polymers.^26^ When the polymers weight ratios in the blend are changed the morphology of thin film is altered. In the following sections, we present results obtained for four different polymer blends, discuss morphologies of corresponding thin films, and compare incorporation of silver nanocubes into such films. The blends chosen were PS/PMMA/P2VP with corresponding weight ratios of 34.5/31.0/34.5% (blend 1), 48.7/23.1/28.2% (blend 2), 30.3/33.3/36.4% (blend 3), and 60/20/20% (blend 4). AFM images of the films obtained for each blend (Fig. 1) reveal micrometer scale isolated domains with 50–100 nm height variations.

### AgNC–polymer blend 1 (34.5/31.0/34.5%, PS/PMMA/P2VP)

**Fig. 2:**
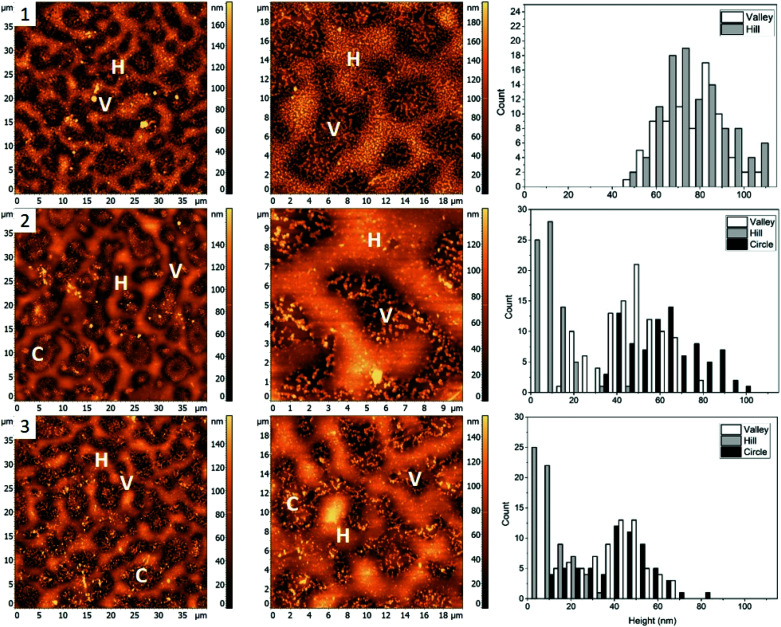
AFM images (left and middle) and corresponding height histograms (right) for a 34.5%/31.0%/34.5% (PS/PMMA/P2VP) blend, (1) with AgNCs deposited, (2) subsequently heated to 109.7 ± 0.3 °C, (3) 122.6 ± 0.5 °C. The three segregated domains labelled with H, C, and V, representing “Hill”, “Circle”, and “Valley” respectively.

The first polymer blend used in the study contained PS, PMMA, and P2VP in 34.5/31.0/34.5% weight ratio (Fig. 1(1)). The three polymers were chosen to have a molecular weight above their entanglement threshold to ensure coherent segmental relaxations for the immiscible polymer domains.^28,29^Fig. 2 shows AFM images of thin films prepared using this blend with silver nanocrystals deposited on top. At room temperature (Fig. 2, row 1) there are two distinct segregated areas seen in the film with ∼50 nm height difference between them, similar to the polymer film without nanocrystals (Fig. 1(1)): “Hill” and “Valley” domains, labelled as H and V in the figure respectively. Lateral dimensions of the domains are on the order of several micrometres, which is sufficiently large to guarantee deposition of each silver nanocrystal on a single type of domain. From the histogram obtained by measuring individual heights for a number of nanocubes (Fig. 2, row 1, right column) we obtained an average height of 77 ± 14 nm and 77 ± 13 nm on H and V domains, respectively. Most of the nanocubes are present as individual particles. Occasional aggregates of nanocubes can be however seen throughout the film, especially in the ‘Valley” domains.

As the sample is heated to ∼110 °C, which is above the glass transition temperatures of PS and P2VP, but below the *T*_g_ of PMMA (Fig. 2, row 2), height of nanocubes on the “Hill” domains is reduced to only 10 ± 7 nm, indicating almost full embedment into that polymer domain (Fig. 2(2), right). A small amount of embedding into the “Valley” domains is also seen (height is measured to be 48 ± 14 nm). Visible aggregation of the AgNCs in this domain is also noticed. In addition to changes in nanocube height, significant morphological changes of the polymer film are notices as well, with appearance of new well defined circular domains, “Circle”, labelled as C. The AgNCs on these domains are 55 ± 18 nm in height. Since *T*_g_ of PS is the lowest of the three polymers used, it is reasonable to expect the largest degree of embedding for nanocubes residing on that polymer, therefore suggesting that the “Hill” domains are in fact PS. Similarly, since P2VP has a *T*_g_ around 105 °C, it is expected that the “Valley” domains are made of P2VP. This is also supported by nanocube aggregation seen in the ‘Valley’ domains, and not the other two and is attributed to the polar nature of P2VP, as no significant aggregation was seen for PS or PMMA homopolymers.^15^ By exclusion, circular domains observed in Fig. 2, second row are identified as PMMA.

When the sample is heated even further, above the *T*_g_ of PMMA as well (Fig. 2, row 3), full embedment into the “Hill” domains is seen, with the final nanocube height of 10 ± 8 nm (Fig. 2(3), right). Approximately halfway embedding into the “Circle” domains was observed (34 ± 15 nm), supporting the idea of the “Circle” domains to be PMMA. Interestingly, no further embedding into the “Valley” domains is seen, as the height of the AgNCs is measured to be 42 ± 14 nm. This is most possibly due to aggregation of the nanoparticles, which reduces the overall nanoparticle surface energy, and thus effectively removing the surface energy as the driving force for embedding and making the embedding process thermodynamically unfavourable. Due to the unfavourable interaction between the predominately non-polar PVP capping agent of silver nanocrystals and the polar P2VP surface, self-association of the nanocubes occurs to minimize this interaction, observed as aggregation in the AFM images.

**Fig. 3:**
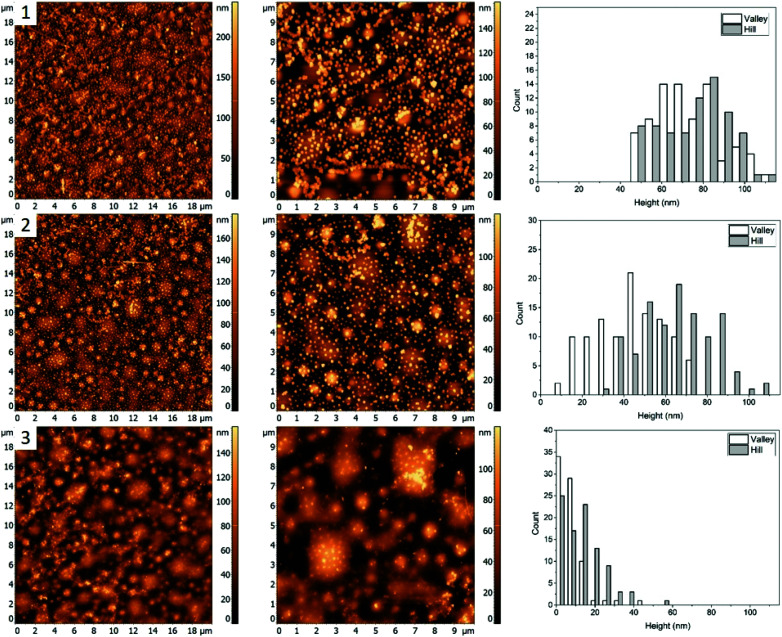
AFM images (left and middle) and corresponding height histograms (right) for a 48.7%/23.1%/28.2% (PS/PMMA/P2VP) blend, (1) with AgNCs deposited, (2) subsequent heating at 97.3 ± 0.3 °C, (3) heating at 120.6 ± 0.5 °C.

### AgNC–polymer blend 2 (48.7/23.1/28.2%, PS/PMMA/P2VP)

Morphology of the PS/PMMA/P2VP film is very sensitive to the weight fraction of constituting polymers. As can be seen in Fig. 1, when the relative content of PS increases by ∼14% the film goes from large ring like chains (Fig. 1, row 1), to smaller hill domains (Fig. 1, row 2). Such high sensitivity reflects the complex nature of inter-molecular forces within the blend. Nanocubes, when deposited on the film are measured to have similar height of 73 ± 19 nm and 68 ± 21 nm, for the “Hill” and “Valley” domains, respectively (Fig. 3, row 1).

As the sample is heated to a temperature which is just below the bulk *T*_g_ of PS (Fig. 3, row 2), a small decrease in height to 44 ± 17 nm for the AgNCs on the “Valley” domains is observed, while for the “Hill” domains the height essentially remains unchanged (67 ± 17 nm). This small amount of embedding even below the bulk *T*_g_ is attributed to the presence of previously observed nanometer scale thin surface layer for PS homopolymers.^9^ This surface layer has increased mobility, effectively resulting in a lower *T*_g_, thus allowing for a small amount of embedding below the bulk *T*_g_ of the polymer. Interestingly, small sections of AgNC aggregates can be seen, which signifies the presence of some P2VP domains.

When the sample is heated well above the *T*_g_ of all three polymers (Fig. 3, row 3), essentially full embedment is observed, with an average height of 8 ± 7 nm and 14 ± 10 nm for the “Valley” and “Hill” domains, respectively. Interestingly, the aggregated nanocrystals are largely undisturbed, with an average height of 65 nm. Thus, even at 120 °C the embedding of aggregated particles is still thermodynamically unfavourable.

### AgNC–polymer blend 3 (30.3/33.3/36.4%, PS/PMMA/P2VP)

**Fig. 4:**
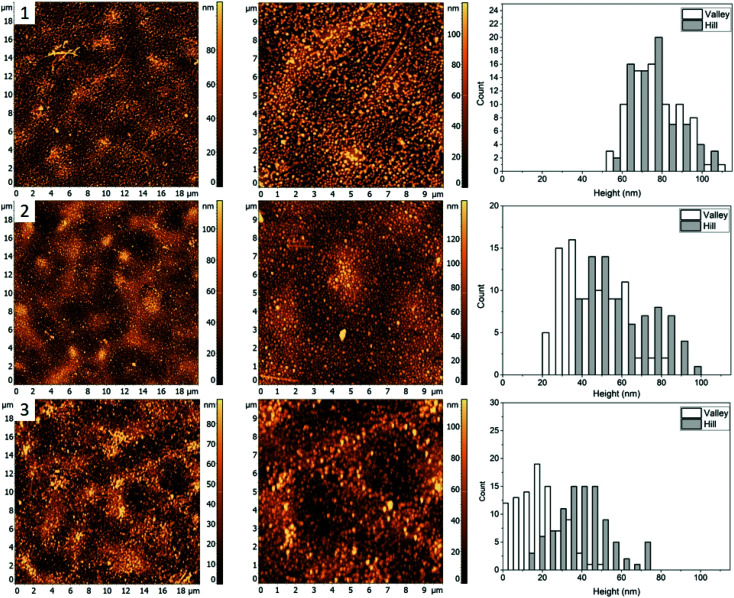
AFM images (left and middle) and corresponding height histograms (right) for a 30.3%/33.3%/36.4% (PS/PMMA/P2VP) blend, (1) with AgNCs deposited, (2) subsequent heating at 99.7 ± 0.3 °C, (3) heating at 122.3 ± 0.4 °C.

For the third mixture, the relative content of PMMA and P2VP in the blend is slightly increased to 33.3 and 36.4% compared to the blend 1. The resulting film topography (Fig. 1, row 3) shares similarities to the first blend (Fig. 1(1)), with two major types of domains forming a semi-circular chain like structure. However, there seems to be a decrease in domain separation, which is especially pronounced after the nanocrystals are deposited (Fig. 4, row 1). Unlike blend 1 (Fig. 2, row 1), however, the circular ring-like structures assumed to be from PMMA are present even before heating. However, due to less visible difference between the circular and the valley – like domains, for this blend we only measured height of nanocrystals on the “Hill” and “Valley” domains and found them to be 78 ± 15 nm and 79 ± 14 nm respectively.

When the sample is heated at just below the bulk *T*_g_ of PS (Fig. 4, row 2), a small degree of embedding is seen, with the average height of the cubes, 46 ± 15 nm for the “Valley” and 59 ± 18 nm for the “Hill” domain. Unlike blend 1, no visible aggregation of nanocrystals is observed. It is possible therefore that P2VP is not exposed at the surface but is rather buried in the film, similar to what was seen for a 33/33/33 (PS/P2VP/PMMA) blend spin casted on a SiO_*x*_ substrate.^26^ Consequently, the valley domains are hypothesized to be PS, thus suggesting inversion of the domain due to a subtle increase in the P2VP and PMMA, while reducing PS from 34.5% (Fig. 2) to 30.3% (Fig. 4).

As the sample is further heated above the *T*_g_ of PMMA (Fig. 4, row 3), near full embedding into the “Valley” domains is seen, supporting the PS hypothesis, with an average height of 19 ± 11 nm. A small amount of embedding was also found for the “Hill” domains with an average height of 40 ± 14 nm, suggesting the “Hill” domains are PMMA. Interestingly, the degree of embedding is much lower than for the blend 2 (Fig. 3, row 3), which had an average height of 8 ± 7 nm and 14 ± 10 nm for the “Valley” and “Hill” domains, respectively, at 120.6 ± 0.5 °C, which is about 2 °C lower. This result suggests either a reduction in the embedding rate, or a new thermodynamic minimum state.

### AgNC–polymer blend 4 (60/20/20%, PS/PMMA/P2VP)

**Fig. 5:**
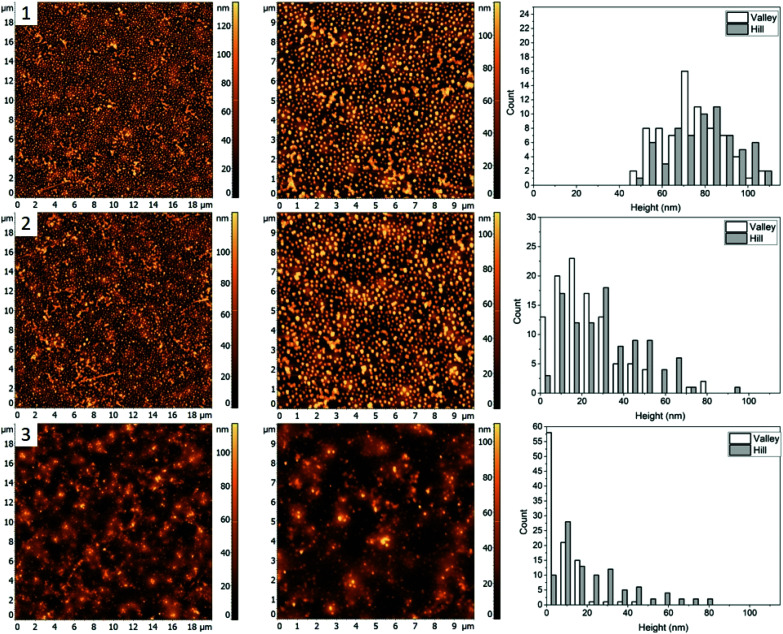
AFM images (left and middle) and corresponding height histograms (right) for a 60.0%/20.0%/20.0% (PS/PMMA/P2VP) blend (1) with AgNCs deposited, (2) subsequent heating at 98.4 ± 0.3 °C, (3) heating at 112.5 ± 0.6 °C.

Fig. 1(4) shows the AFM images for a 60/20/20% (PS/PMMA/P2VP) blend. Morphology of this blend is similar to the 48.7%/23.1%/28.2% blend (Fig. 1, row 2) with well-defined circular domains, except in this case the domains are considerably smaller. The height of silver nanocrystals is found to be 76 ± 18 nm and 70 ± 17 nm for the “Hill” and “Valley” domains, respectively (Fig. 5(1)). Some nanocrystals aggregate, suggesting the presence of P2VP at the surface.

When the sample is heated just below the bulk *T*_g_ of PS (Fig. 5, row 2), a small amount of embedment is seen in the “Valley” domains, with a decrease in average height to 54 ± 19 nm. However, the height of the AgNCs on the “Hill” domains is unaffected at 70 ± 18 suggesting the “Valley” domains to be PS and the “Hill” domains – PMMA.

When the sample was further heated to just below the bulk *T*_g_ of PMMA (Fig. 5, row 3), full embedment into the “Valley” domains is observed with an average height of 8 ± 8 nm, indicating this to be PS. Interestingly, near full embedment into the PMMA domains is seen as well, with a height of 25 ± 19 nm. This likely suggests different kinetics for this blend in comparison to 30.3/33.3/36.4% (Fig. 4), which further suggests possible surface confinement effects, causing a reduction in embedding rate.

### Embedding summary

The glass transition dependence of AgNC embedment enabled the identification of discrete polymer domains within the PS/PMMA/P2VP ternary polymer blends from the relative heights of the AgNCs at various temperatures; since AgNCs would only embed when the polymer is above its glass transition temperature (*T*_g_). This makes possible the selective embedment of the AgNCs into the polymer domains and can act as a tool to identify the polymers in the mixture. A summary of the temperature dependence of the embedding of the nanocubes into each polymer domain can be found in Table 1. Below is a brief overview on the selective embedment into the immiscible ternary polymer blends.

**Table 1:** Summary of the AgNCs height on each discrete polymer domain for all polymer blends, with the corresponding temperature (*T*_r_ is room temperature)

Blend (PS%/PMMA%/P2VP%)	Temperature (°C)	*H*_Hill_/(nm)	Polymer	*H*_Valley_/(nm)	Polymer	*H*_Circle_/(nm)	Polymer
Blend 1: 34.5/31.0/34.5	*T*_r_	77 ± 14	PS	77 ± 13	P2VP		
	109.7 ± 0.3	10 ± 7		48 ± 14		55 ± 18	PMMA
	122.6 ± 0.5	10 ± 8		42 ± 14		34 ± 15	
Blend 2: 48.7/23.1/28.2	*T*_r_	73 ± 19	PMMA	63 ± 21	PS		
	97.3 ± 0.3	67 ± 17		44 ± 17			
	120.6 ± 0.5	14 ± 10		8 ± 7			
Blend 3: 30.3/33.3/36.4	*T*_r_	78 ± 15	PMMA	79 ± 14	PS		
	97.7 ± 0.3	59 ± 18		46 ± 15			
	122.3 ± 0.4	40 ± 14		19 ± 11			
Blend 4: 60.0/20.0/20.0	*T*_r_	76 ± 18	PMMA	70 ± 17	PS		
	98.4 ± 0.3	70 ± 18		54 ± 19			
	112.5 ± 0.6	25 ± 19		8 ± 8			

For blend 1 it was determined that the “Hill” domain was PS, the “Valley” domain was P2VP and the “Circle” domain was PMMA. The aggregation of silver nanocrystals on the “Valley” domain supports identification of the domains as P2VP since AgNC aggregation is uncharacteristic for PMMA and PS homopolymers.^9^ Essentially full embedment of the AgNCs into the “Hill” domain was observed at 109.7 ± 0.3 °C, confirming it as PS. The AgNCs on the PMMA domain embedded about halfway at 122.6 ± 0.5 °C, while the AgNCs only embedded slightly into the P2VP domain at the same temperature.

The PMMA domain was only apparent after heating of the sample to 109.7 ± 0.3 °C, suggesting a change in interfacial energy of the film, possibly due to the deposition of the AgNCs. This caused polymer re-arrangements to obtain a new thermodynamically favourable state. Interestingly, the PMMA domains become less apparent when the sample is heated to 122.6 ± 0.5 °C, potentially due to additional polymer re-arrangements.

For blend 2 it was determined, that the “Hill” domain was PMMA and the “Valley” domain was PS. Unlike the first blend, essentially full embedment was observed for AgNCs on both the PS and PMMA domains heated to 120.6 ± 0.5 °C (Fig. 3). A small amount of particle aggregation was observed indicating presence of P2VP at the surface, which suggests that the majority of the polymer was below the surface, predominately interacting with the polar glass surface. The topology of the sample is very different from the first blend, with small circular instead of the larger chain-like domains (Fig. 1), which is an indication of the tunability of the ternary blend.

For the third blend used it was determined that the “Hill” domain was PMMA and the “Valley” domain was PS, similar to blend 2. Near complete embedment was observed for AgNCs on the PS domain and less than half for the PMMA domain heated to 122.3 ± 0.4 °C. The reduction of embedding into the PS and PMMA domains with respect to the first blend (Fig. 2) was likely due to, (a) change in the interfacial surface energy caused by altering the composition of the solution or (b) reduction in the embedding kinetics. The topology of the sample appears similar to the 34.5/31.0/34.5% blend (Fig. 1) except the PMMA and P2VP domains have inverted.

For the fourth blend used the “Hill” domain was identified as PMMA and the “Valley” domain as PS (Fig. 5). Full incorporation of the AgNCs was observed for the PS domain and near full embedment was determined for the PMMA domain heated at 112.5 ± 0.6 °C. This is comparable to the 48.7/23.1/28.2 blend (Fig. 3) and is greater than for the 30.3/33.3/36.4 (Fig. 4) blend heated to ≈120 °C, which further signifies a blend dependent reduction in embedding rate. The topology of the sample (Fig. 5) lends characteristics from the blends 2 and 3 since it possesses similar circular domains as found in sample 48.7/23.1/28.2 (Fig. 3), with chain like domains similar to the 30.3/33.3/36.4 (Fig. 4) blend.

### Surface confinement effects

One of the advantages of using silver nanocubes as fillers in nanocomposite materials is the ability to monitor the process of nanoparticle incorporation in real time. Plasmonic spectral features of such nanocubes are very characteristic and sensitive to the embedment process, with nanometer scale precision.^9^

**Fig. 6:**
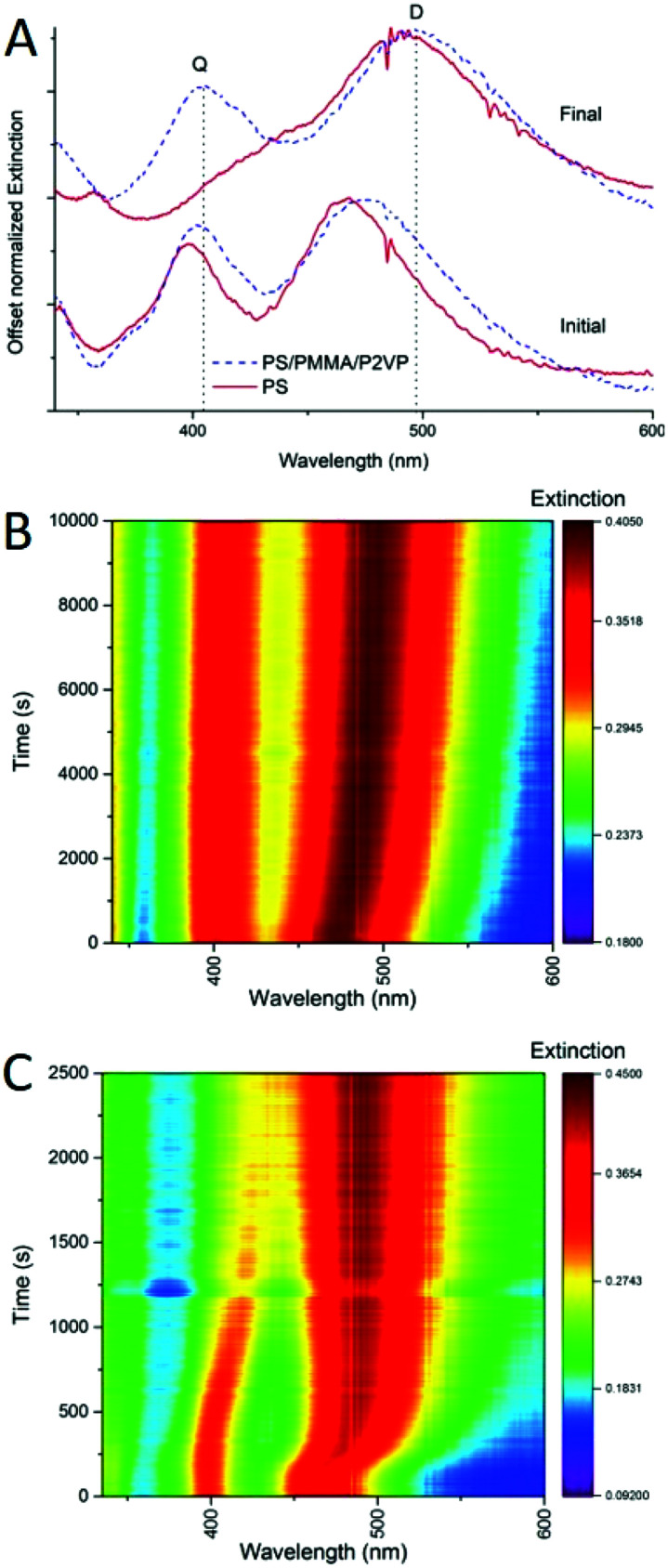
(A) UV-vis spectra from the heating experiments for AgNC–polymer nanocomposites, (blue) a 60/20/20 (PS/PMMA/P2VP) polymer blend and a homopolymer PS (red) initially at *t* = 0 (solid), and the final spectrum (dashed). (B) UV-vis spectral heatmap for the 60/20/20 blend-AgNC heating experiment. (C) UV-vis spectral heatmap for the PS homopolymer–AgNC heating experiment.

In the previous study, we explored this sensitivity to investigate incorporation of silver nanocrystals into homopolymer thin films of PS, and PMMA.^9^ In addition to embedding depth control we were able to determine the diffusion constants for the nanocrystals incorporation into thin films as well as the activation energies of embedment. Similarly, the spectroscopic response of the nanocrystals can be used to monitor the incorporation process into films made of a blend of polymers. For example, Fig. 6 shows extinction spectra (A) and spectral maps (B and C) for thermally activated silver nanocube incorporation into homopolymer film of PS and into a heteropolymer film made of PS/PMMA/P2VP 60/20/20% blend. Before heating there are two main extinction peaks seen (Fig. 6A), named dipolar (D) and quadrupolar (Q), known to be sensitive to the substrate and surrounding environment refractive indices respectively.^9^ When heated above the glass transition temperature of PS, sufficient to initiate nanocube embedding, the spectral signatures change significantly, with the red-shifting of the dipolar peak (Fig. 6A). This is well resolved in the spectral maps (Fig. 6B and C), for the blend and PS. Depth of embedding is correlated with the spectral shift.^9^ At the same time, details of spectrally observed embedment for the polymer blend are quite different form the homopolymer PS film. First, the initial spectral shift of the D peak for the blend is observed over a longer period of time as compared to the PS only film (Fig. 6B*vs.*Fig. 6C). Partially, this is due to a slightly lower temperature used for the blend. Also, in the blend only 60% of the film is PS, and as such at most ∼60% of the cubes would embed and induce spectral change at the temperature used. The remaining ∼40% of the cubes remain on top of the film with spectral signatures unchanged. Second, the D peak position for the two samples is different initially and the peak is broader for the blend than for PS (Fig. 6A), due to the difference in the refractive index of the films, which influences the D initial position. Specifically, of the three polymers PMMA has the lowest refractive index (*n* = 1.497 at 486.1 nm),^30^ and PS has the highest (*n* = 1.599 at 486.1 nm).^30^ The refractive index of P2VP hasn't been thoroughly studied, but is believed to be around 1.5 over the visible range.^31,32^ Since the UV-vis measurement is from the ensemble of all the cubes on each polymer, the resulting D and Q peaks are the superposition, leading to broader peaks than for nanocubes deposited on a single polymer (Fig. 6A). Finally, the PS homopolymer supported sample shows near full embedment of the AgNCs, indicated by the overlapping of the D and Q peaks (Fig. 6A, final red spectrum), while for the blend almost no shift in the Q mode is seen, and about a 25 nm shift in the D mode is observed (Fig. 6A, final, blue spectrum). Comparing the heat maps between these samples (Fig. 6B and C) it is clear that the rate of embedding is drastically different. This could be due to the slight temperature difference used in the two experiments. For the AgNC–PS nanocomposite essentially full embedment is observed at 2000 s (Fig. 6C). This is in stark contrast to the blend, where the D mode continues to slowly shift even at 10 000 s, and Q mode only shifts 4 nm in total (Fig. 6B). Overall, this analysis tells us that spectral behavior of nanocubes upon embedding into polymer blend can be of use for controlling the embedding depth.

**Fig. 7:**
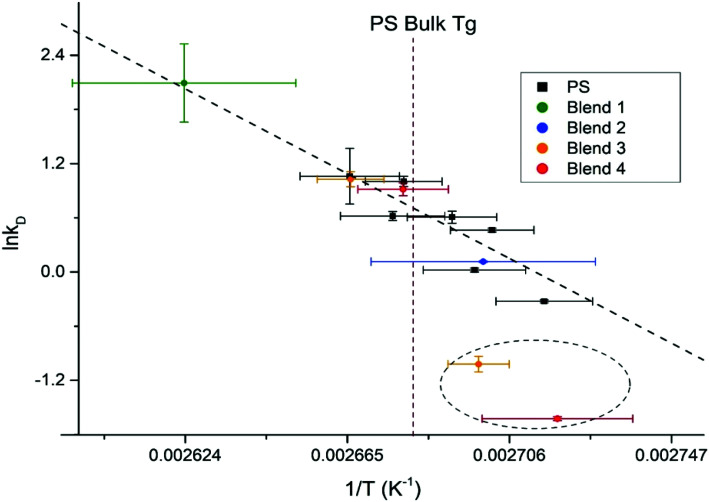
Arrhenius plot for homopolymer PS films, with the PS kinetic data from the ternary polymer overlaid. The bulk *T*_g_ of PS is outlined as the maroon dashed line.

To perform a temperature independent comparison of embedding dynamics of silver nanocrystals into homo *vs.* hetero polymer films we further analyzed Arrhenius dependences of diffusion constant *k*_D_ into PS^9^ (Fig. 7, black squares) and into PS domains within the blend (Fig. 7, colored circles) thin films. Diffusion of silver nanocrystals into PS homopolymer films appears to follow the Arrhenius behavior, showing a good linear fit (indicated by the dashed line) as we demonstrated recently.^9^ It can be seen that such linear dependence is observed even at temperatures slightly below the bulk PS glass transition temperature, shown as vertical dashed line. This indicates that the *T*_g_ of the top most layer is slightly lower than the bulk *T*_g_, as we showed previously.^9^ The hetero polymers fairly good agreement with the PS homopolymer Arrhenius plot as can be concluded from all four polymer blends (Fig. 7, points on the left side of line, indicating *T*_g_) at temperatures above the bulk *T*_g_ for PS diffusion, which suggests little to no change in the surface energy of the system caused by the addition of the immiscible polymers. At the same time, for the hetero polymer film as the temperature drops below the bulk PS glass transition temperature, for at least two blends (30.3/33.3/36.4% and 60.0/20.0/20.0%), show embedding behavior deviating significantly from the linear trend (two points circled). Therefore, it appears that changes in polymer film composition primarily affects properties of the topmost layer, responsible for the nanocrystal embedding below the *T*_g_ this is in agreement with literature results obtained using spherical polymer particles.^33^ The paper showed that varying the size of the particle does not change the bulk *T*_g_ of the polymer, but it does drastically increase the specific heat capacity, which is represented as a thin layer on top of the bulk polymer.^33^ Another study looked at the reduction in *T*_g_ for polymer spheres capped with silica, and found that the confinement effect seen for spheres is not due to the shape of the polymer, but rather the interfacial surface energy difference from the free surface and the bulk polymer.^34^ By modifying the composition of the polymer blend, the surface free energy is changing,^24,35^ which likely is the cause for the enhancement of the surface confinement effects.

## Conclusions

This work develops a technique to fabricate and controllably optimize nanoparticle-blended polymer nanocomposite functional materials, based on the thermally activated irreversible embedment of AgNCs into polymer surfaces. Immiscible polymer blended films were produced through a simple spin coating approach, forming defined polymer domains, driven through self-association of the polymers. Utilizing the temperature selectivity of the embedding process AgNCs were selectively embedded into individual polymer domains, within immiscible ternary polymer blends. This allowed us to locate specific polymers within the blends. As the AgNCs embed into a polymer, the effective refractive index increases, causing a shift in the peak positions observed in a UV-vis spectrum. This gives a sensitive way to monitor the embedding of the AgNCs in real-time, allowing the determination of the dynamics for the embedding process. Based on these UV-vis measurements, surface confinement of PS was observed for some polymer blends; however, the bulk dynamics was not affected. This work emphasizes a controllable technique to produce, characterize and optimize nanoparticle-polymer nanocomposite materials, while monitoring the embedding process in real time. This is of interest in fields that require fine control over nanoparticle incorporation within segregated polymer domains, such as polymer blends and copolymers, which is of particular interest to optoelectronic, photocatalysis, and photovoltaics devices. This work also brings interesting insight into polymer dynamics within polymer blends.

## Conflicts of interest

There are no conflicts to declare.

## Supplementary Material
